# Retraction: Green Tea Catechins Reduce Invasive Potential of Human Melanoma Cells by Targeting COX-2, PGE_2_ Receptors and Epithelial-to-Mesenchymal Transition

**DOI:** 10.1371/journal.pone.0210345

**Published:** 2018-12-31

**Authors:** The PLOS ONE Editors

Following publication of this article [1], concerns were raised about similarities involving two figure panels.

The second and third panels in Fig 5D (Cay10580, 0.1 μM and 1.0 μM panels) are similar. The University of Alabama at Birmingham confirmed that the original data and records needed to clarify the conditions represented in these two panels are not available. Thus, the quantification data shown in the accompanying bar graph, and the conclusion regarding a concentration-dependent effects of Cay10580 on cell migration, are not supported.

Fig 2A (Hs294t, 10 μg/ml EGCG) in [1] is similar to Fig 1B (Hs294, 0 μM Berberine) in [2], even though the panel in question is used to represent different experimental conditions in the two articles. The University of Alabama at Birmingham confirmed that original data and records are not available to clarify the identity of the cells and treatment conditions in the reported experiments. The *Carcinogenesis* article [2] was retracted in 2018 [3].

Following a joint investigation by the Birmingham VA Medical Center and the University of Alabama at Birmingham, the institutions requested retraction of this article, as the conclusions could not be supported by available data. In line with the institutions’ recommendation, *PLOS ONE* Editors retract this article based upon the unavailability of original data and records and the ambiguous identification of samples and treatments.

The authors did not comment on the retraction decision.
